# Efficacy of Repeated Low-Level Red Light (RLRL) Therapy in Managing Childhood Myopia: A Systematic Review and Meta-Analysis

**DOI:** 10.3390/jcm14010083

**Published:** 2024-12-27

**Authors:** Maria Sobol, Jacek Pniewski

**Affiliations:** 1 Department of Biophysics, Physiology and Pathophysiology, Medical University of Warsaw, Chałubińskiego 5, 02-004 Warszawa, Poland; 2 Faculty of Physics, University of Warsaw, Pasteura 5, 02-093 Warszawa, Poland; j.pniewski@uw.edu.pl

**Keywords:** myopic children, myopia control, RLRL therapy, meta-analysis, systematic review

## Abstract

**Objectives**: As Repeated Low-Level Red Light (RLRL) therapy is becoming increasingly prevalent in clinical practice, mainly in the Far East, largely due to its child-friendly nature and the feasibility of home use, this study aims to conduct a systematic review and meta-analysis to evaluate the efficacy of RLRL therapy in managing childhood myopia, specifically in relation to axial length (AL) and spherical equivalent refraction (SER), across a larger group of children aged from 6 to 16 years. **Methods**: A systematic literature search was performed using PubMed, Scopus, and Web of Science to access relevant databases and to locate outcome studies. Eligibility criteria included publication type, participant characteristics, and outcomes report. As appropriate, data analysis was conducted using either a random or fixed effects model. **Results**: Ten articles were included in the final analysis. All the studies included in the analysis were conducted in China and most of them is shortened to one year follow-up time. The mean difference in change of AL between the study and control groups was −0.33 mm with confidence levels ranging from −0.42 to −0.25 mm. The mean difference in change of SER between the study and control groups was 0.63 D with confidence levels ranging from 0.42 to 0.85 D, which was found to be statistically significant (*p* < 0.001). The mean difference in AL change at t = 6 months for the RLRL and SVS groups was 0.00 mm (95% CI: −0.10 to 0.10 mm) and 0.23 mm (95% CI: 0.15 to 0.32 mm) respectively. At t = 12 months mean difference in AL change for the RLRL and SVS groups was −0.01 mm (95% CI: −0.16 to 0.13 mm) and 0.35 mm (95% CI: 0.20 to 0.50 mm) respectively. The mean difference in SER change at t = 6 months for the RLRL and SVS groups was 0.18 D (95%CI: −0.03 to 0.39 D) and −0.48 D (95% CI: −0.69 to −0.27 D, respectively. At t = 12 months the mean difference in SER change for RLRL and SVS groups was 0.05 (95% CI −0.31 to 0.42 D), and −0.73 D (95% CI: −1.08 to −0.37 D), respectively. **Conclusions**: The results of the meta-analysis indicate that myopic children who received RLRL therapy in addition to standard myopia management demonstrated a slower progression of myopia compared to the control group. These findings suggest that RLRL therapy may be an effective novel adjunctive treatment for myopia controls.

## 1. Introduction

Myopia is a major concern in the public health context [1], due to its increasing prevalence globally [2,3,4] and the associated risk of conditions that can lead to blindness, such as myopic maculopathy, glaucoma, retinal detachment, and certain types of cataract [5,6,7,8,9]. Projections indicate that more than half of the world’s population will be myopic by 2050. What is more, the increasingly early occurrence of myopia in children and its consequent faster progression is contributing to an epidemic of potentially blindness-causing high myopia. Myopia also reduces the learning efficiency of school children and young adults, and affects their school admission [10]. Although certain models to predict the development of myopia in children were developed, there is an urgent need for efficient methods of myopia progression control [11,12,13,14].

Several current studies for myopia were proposed, which include low-concentration atropine [15,16,17,18], orthokeratology [18,19,20], defocus incorporated multiple segments (DIMS) spectacle lenses [21,22], and bi-, multifocal or special soft contact lenses [23,24,25,26]. However, these treatments are only successful to a certain extent; they rarely stop progression, and they are not effective in some children [27,28]. Furthermore, even though these methods can delay the progress of myopia, some adverse events can still occur, such as myopia rebound, photophobia, and allergic reactions [1].

We probably do not currently have strategies for preventing myopia development that stop its progression in all children. Still, the most important approach is encouraging children to spend more time outdoors and educating them on near-work considerations such as increased viewing distance and frequent breaks [29].

Recent studies claim that repeated low-level red-light therapy (RLRL), which could be also combined with other methods, can effectively delay the progress of myopia [30,31,32,33,34,35,36,37,38,39,40,41,42,43]. Although current suggestions are not to combine RLRL therapy with other methods, as there is probably no statistical difference compared to RLRL, the authors still test such combinations with some success in particular groups [44,45]. Red light refers to the visible-light wave range 600–700 nm. This kind of light influences mitochondrial functions and is used in the clinical treatment of some diseases; for example, in dermatology [46]. Even though the complete mechanisms of action of all mentioned methods are still under investigation, the published studies have shown effectiveness in controlling myopia progression in children.

Despite the reported successes in RLRL therapy, several contraindications may limit its usefulness. It is not recommended in patients with a history of photosensitivity, macular diseases, dry eye, uveitis, optic nerve pathologies, and many others, although there is still a limited number of publications that analyze these aspects of safety. 

Even though the aforementioned red-light therapies use low-intensity light, the use of light is debated, regarding safety. Some authors claim that retinal illumination exceeds safety limits [47], or raise questions about safety [48]. Others present results confirming no considerable changes in the eye after 1-year’s therapy but also note some changes in the morphology of photoreceptor outer segments and relative reflectance of the ellipsoid zone, which require further investigation [43]. Chen Y. et al. analyzed 20 studies encompassing 2380 participants, for up to 24 months of therapy, and found no irreversible abnormalities or permanent vision loss [49]. It should also be noted that the appropriate ethical committees approved all studies, and some of them are listed in the clinical trials database. Although most of the studies are conducted in China, there are currently ongoing studies on myopia progression at the Univ. of California, USA [50], and Univ. of Houston [51], as listed in the ClinicalTrials.gov.

According to the authors, irradiation frequency should not exceed 2 times a day, each treatment time should not be longer than 3 min, and the interval between successive treatments should be a minimum of 4 h.

In this paper, we conducted a systematic review and meta-analysis based on a comprehensive review of the existing literature to summarize and quantify the data supporting the use of RLRL in the treatment of myopia.

## 2. Materials and Methods

**Figure 1:**
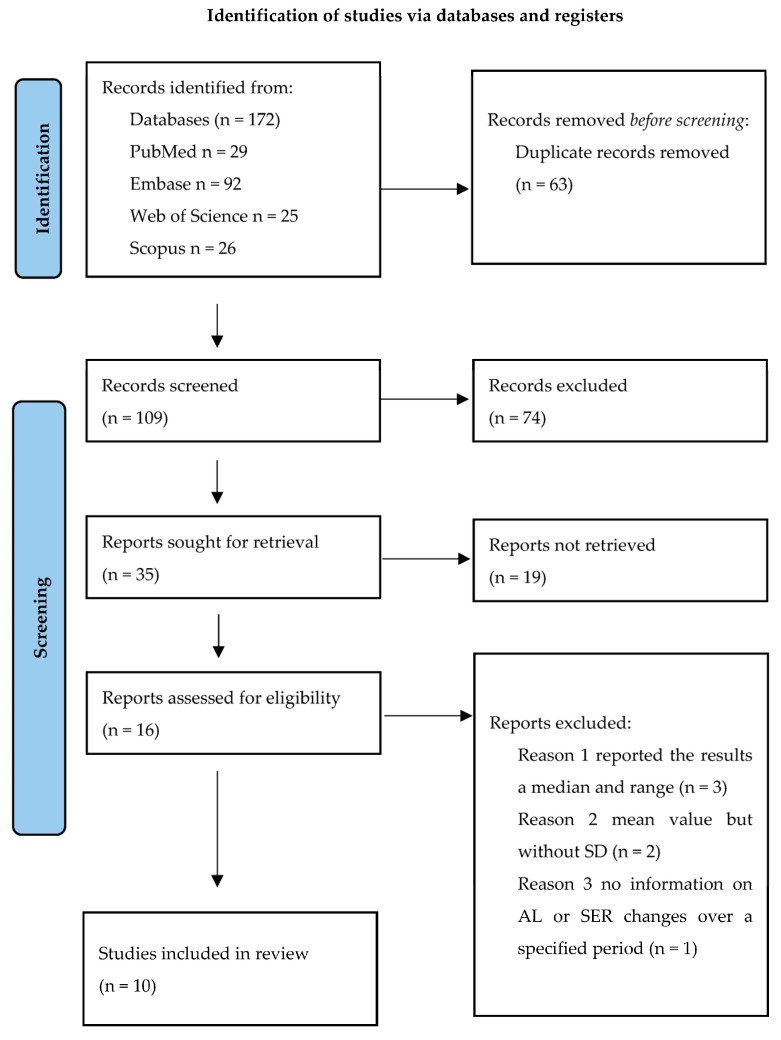
Flow chart for inclusion of studies.

Studies included in this research were selected from a systematic search of the literature in Scopus, Web of Science, and PubMed. Studies published up until 29 September 2024 were considered. The inclusion criteria were as follows: original papers, retrospective and prospective papers, randomized control trials, human studies, and English language. The screening of the results was based on the following phrases: “repeated low-level red-light therapy” OR “RLRL”, “myopia” OR “nearsightedness”, and “children”. For the analysis, the following were excluded: studies that only reported the results as (1) median and range, median and interquartile range (IQR) of axial length (AL), and spherical equivalent refraction (SER) or mean value but without standard deviation (SD) or confidence interval (CI), or (2) studies that provided no information on AL or SER changes over a specified period. (3) Additionally, studies involving atropine therapy in children were excluded (Figure 1). Case reports, unpublished reports, and abstracts were not considered. The authors were not contacted. Two reviewers, MS and JP, assessed each abstract and full text for potential inclusion, and reached a consensus for the articles to be included in the final review.

### 2.1. Study Selection

The systematic review was conducted using the PRISMA guidelines [52,53]. Specific requirements are listed below:

RLRL and SVS groups: children aged from 6 to 16 years with a confirmed clinical diagnosis of myopia, with SER ≤ −0.5 D when ocular accommodation is relaxed [54], astigmatism of 1.5 D or less, and anisometropia of 1.5 D or less, and 10 to 21 mmHg intraocular pressure, who did not receive another myopia therapy except for wearing standard corrective spectacles, without the presence of ocular or systemic diseases such as strabismus, amblyopia, and cardiac respiratory illness. The children were randomly assigned to RLRL or SVS groups.

Limits used: human-subject studies published in English.

Timing: studies published up to and including 29 September 2024.

### 2.2. Statistical Analysis

Statistical analysis was performed using the software package, TIBCO Software Inc. (Santa Clara, CA, USA), Statistica® (data analysis software system), version 13.6.0 (https://www.tibco.com). The Q test was used to test heterogeneity, and I^2^ statistics were calculated to quantify and evaluate the heterogeneity (low: 25–50%, moderate: 50–75%, and high >75%). Since heterogeneity (I^2^ statistics) exceeded 93%, and 96% for AL and SER, respectively, between the RLRL and SVS groups, a random effects model was used for the analysis. For the mean differences calculated separately for the RLRL and SVS groups at time points t = 6 months and t = 12 months, a fixed-effects model was used, as heterogeneity exceeded 0%. The mean differences were reported with 95% CI. Forest plots were generated to showcase the differences between the RLRL and SVS groups for AL and SER parameters and the corresponding 95% CIs for each study, as well as overall estimates. To assess the stability of the plotted results, sensitivity analysis was conducted by excluding each study at a time. To assess for publication bias, Egger’s and Begg’s tests were conducted. The subgroup analysis was provided to find possible reasons for the high heterogeneity level. Moreover, the trim-and-fill method for publication bias was performed, to estimate potentially missing studies.

## 3. Results

The search strategy identified 172 articles in the PubMed, Scopus, and Web of Science databases. Ten studies were selected and hence included [30,31,32,33,34,35,36,37,38,39].

Table 1 summarizes the characteristics of the included studies. Both the RLRL and SVS groups included children with routine treatment for myopia (all participants wore single-vision distance spectacles (SVS) throughout the study). Besides wearing SVS lenses, the study group underwent RLRL treatment. All children in the SVS group were age-matched with the RLRL group. Children who had ocular diseases such as strabismus, binocular vision abnormalities, other ocular abnormalities in either eye, history of ocular surgeries (e.g., cataract, refractive or intraocular surgery), who underwent myopia control treatment, and children with systemic diseases e.g., endocrine, cardiac and respiratory diseases, were excluded from the study. The participants were randomly assigned to the study group (children who, besides wearing SVS lenses, had undergone RLRL therapy) or the control group. All the studies included in the analysis were conducted in China, and the data were collected from children between 2019 and 2023. Table 2 presents details of the devices used for RLRL therapy in the papers listed in Table 1.

In the meta-analysis on the comparison of change in AL and SER values between the RLRL and SVS groups, 1648 children were included (839 in the study group and 785 in the SVS group). The age range of the patients was from 6 to 16 years. The study sample sizes varied from 11 to 139 patients.

In all studies [30,31,32,34,35,36,37,38,39] except Dong J. et al. [33], only the data from a single eye were analyzed. Jiang Y. et al., Xiong R. et al., Zhou L. et al., Chen H. et al., Yan X. et al., Wen Z. et al., and Cao K. et al. analyzed data from the right eye, whereas Xiong F. et al. analyzed data from the left eye, He X. et al. utilized data from the more myopic eye, while Dong J. et al. used the mean values derived from both eyes.

**Figure 2:**
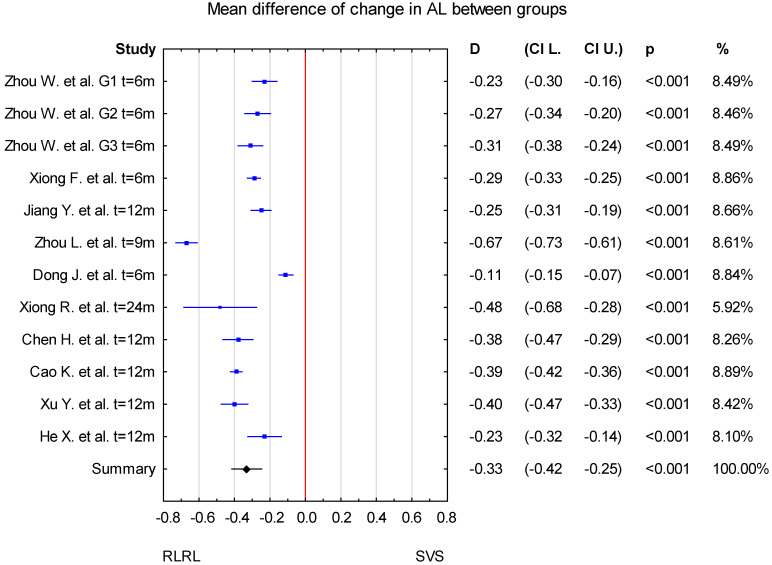
Forest plot of mean difference of change in AL between the RLRL group and SVS group, with *p*-value indicating level of statistical significance. The size of the box represents the point estimate for each study in the forest plot, and is proportional to that study’s weight-estimate contribution to the summary estimate. Horizontal lines represent 95% CI [31,32,33,34,35,36,37,38,39].

At first, for the analysis, we selected data corresponding to the longest reported follow-up time from each included publication. As a result, the mean difference in change in AL between the RLRL and SVS groups was −0.33, with confidence levels ranging from −0.42 to −0.25 mm. The result is statistically significant at *p* < 0.001 (Figure 2).

**Figure 3:**
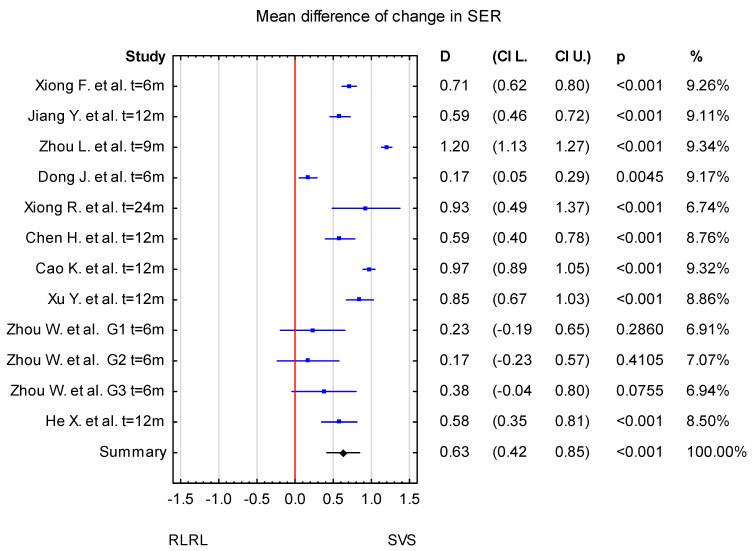
Forest plot of mean difference of change in SER between the RLRL group and SVS group, with *p*-value indicating level of statistical significance. The size of the box represents the point estimate for each study in the forest plot and is proportional to that study’s weight-estimate contribution to the summary estimate. Horizontal lines represent 95% CI [30,31,32,33,34,35,36,37,38,39].

The mean difference in change in SER between the RLRL and SVS groups was 0.63 D, with confidence levels ranging from 0.42 D to 0.85 D. The result is statistically significant at *p* < 0.001 (Figure 3).

### 3.1. Risk-of-Bias Assessment

**Figure 4:**
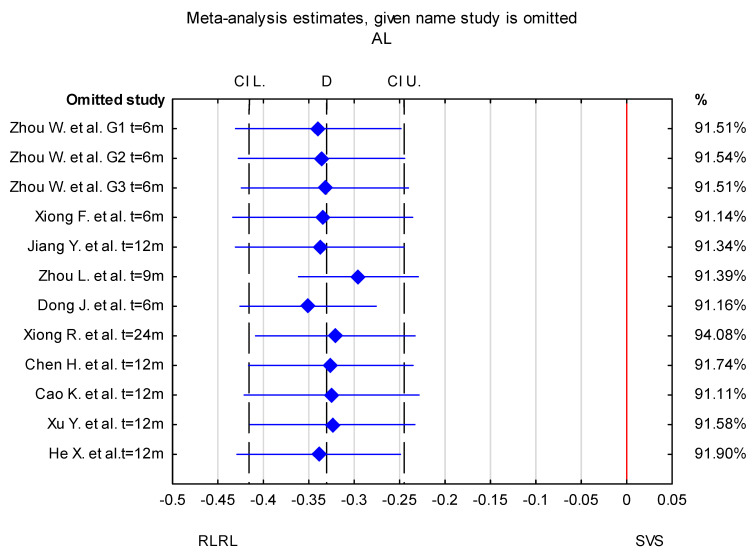
Sensitivity analysis for the effect of individual studies on the pooled difference in AL with confidence intervals. The named study on the Y-axis is omitted from the analysis, to assess the effect it has on the overall results. G1–G3—different power levels (G1—0.37 mW, G2—0.60 mW, and G—1.20 mW) [30,31,32,33,34,35,36,37,38,39].

**Figure 5:**
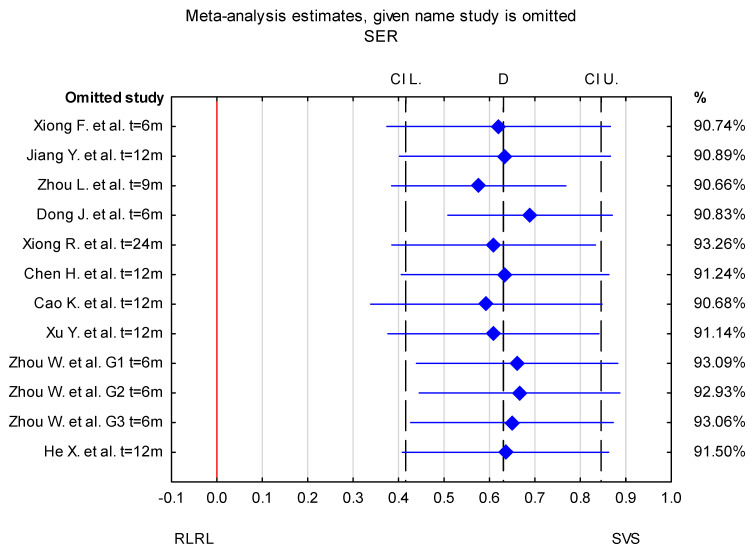
Sensitivity analysis for the effect of individual studies on the pooled difference in SER with confidence intervals. The named study on the Y-axis is omitted from the analysis, to assess the effect it has on the overall results. G1–G3—different power levels (G1—0.37 mW, G2—0.60 mW, and G—1.20 mW) [30,31,32,33,34,35,36,37,38,39].

Overall, in the forest plot for the RLRL vs. SVS group, the studies denoted lower values of change in AL for the study group and less progression of SER. The confidence intervals in the studies by Zhou L. et al. [32] and Dong J. et al. [33] differ from those in the other studies included in the analysis for both AL and SER. The mean difference in AL change between the RLRL and SVS group in a study by Zhou L. et al. [32] is higher compared to other included studies, whereas the value reported by Dong J. [33] et al. is lower. The result of the sensitivity analysis showed that the change in AL mean differences between the RLRL and SVS groups varied from −0.35 mm (95% CI −0.43 to −0.28) when the Dong J. et al. study was excluded, to −0.30 mm (95% CI −0.36 to −0.23 mm) when the Zhou L. et al. [32] study was excluded. For the change in SER, the mean differences between the RLRL and SVS groups varied from 0.68 D (95% CI from 0.51 to 0.87) when the Dong J et al. [33] study was excluded, to 0.58 D (95% CI from 0.39 to 0.76 D) when the Zhou et al. [32] study was excluded. This indicates that the stability of mean differences between the RLRL and SVS groups was not influenced by a single study (Figure 4 and Figure 5).

Furthermore, the result of Egger’s test (*p* = 0.800 for AL and *p* = 0.081 for SER in the RLRL vs. SVS group) also indicated that there was minimal potential for publication bias, which was also consistent with Begg’s test (*p* = 0.652 and *p* = 0.624, respectively).

### 3.2. Subgroup Analysis

**Figure 6:**
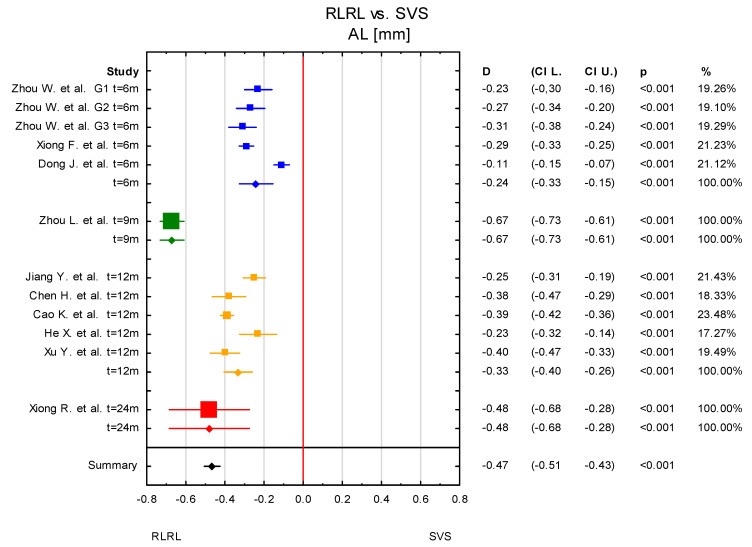
Subgroup analysis according to time of observation. G1–G3—different power levels (G1—0.37 mW, G2—0.60 mW, and G—1.20 mW) [30,31,32,33,34,35,36,37,38,39].

**Figure 7:**
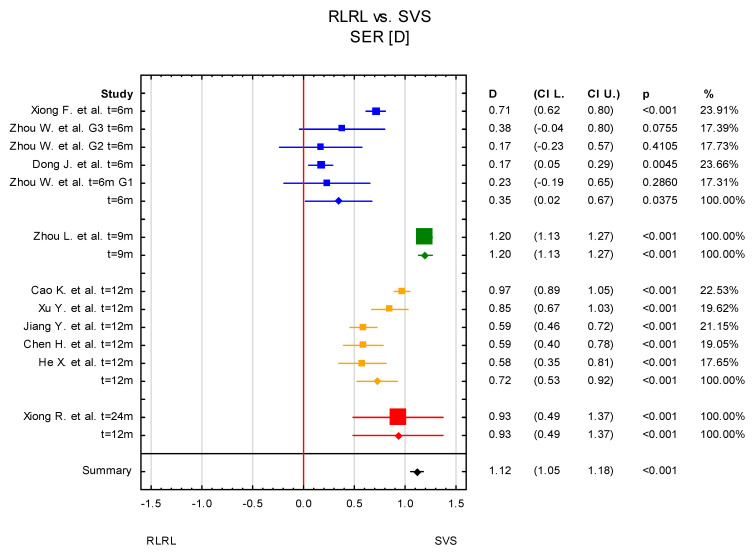
Subgroup analysis according to time of observation. G1–G3—different power levels (G1—0.37 mW, G2—0.60 mW, and G—1.20 mW) [30,31,32,33,34,35,36,37,38,39].

Since the heterogeneity among studies was high, a subgroup analysis for the RLRL vs. SVS group was conducted to try to find possible reasons for the variability of the results. The different time of treatment was considered for the subgroup analysis. In three publications (five results, as Zhou W. et al. [38] considered three groups according to different powers of light sources) which were included in the meta-analysis, data were given after 6 months of undergoing RLRL therapy, in five after 12 months, one after 9 months (Zhou L. et al. [32]) and one after 24 months (Xiong R. et al. [34]). In the subgroup containing three publications (6 months of RLRL therapy) as well as in five publications (12 months of treatment), the overall result was statistically significant. Statistically significant results were also reported for the two subgroups containing Zhou L. et al. [32] and Xiong F. et al. [30], individually (Figure 6 and Figure 7). The subgroup analysis can suggest that the time of providing RLRL therapy impacted overall meta-analysis results. However, it should be noted that two of the subgroup analyses contained only one study, and thus the true effect is hard to establish.

### 3.3. Trim-and-Fill Method

**Figure 8:**
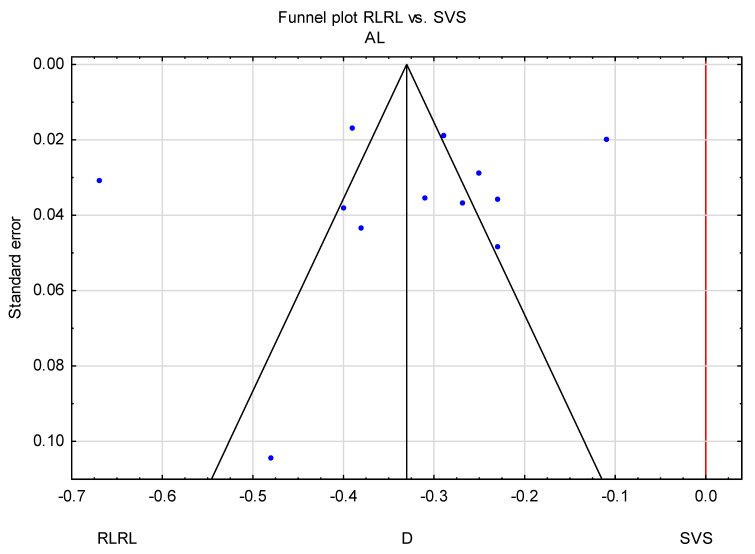
Funnel plot of comparison between the RLRL and SVS groups.

**Figure 9:**
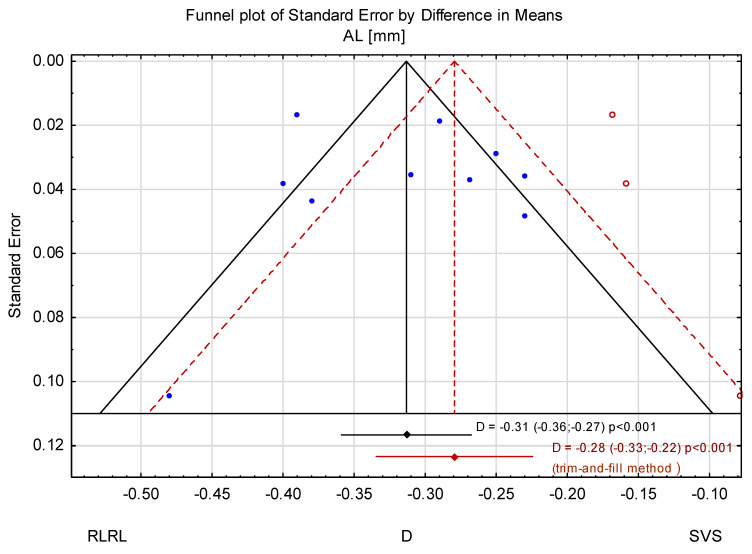
Funnel plot before and after applying the trim-and-fill method (AL).

**Figure 10:**
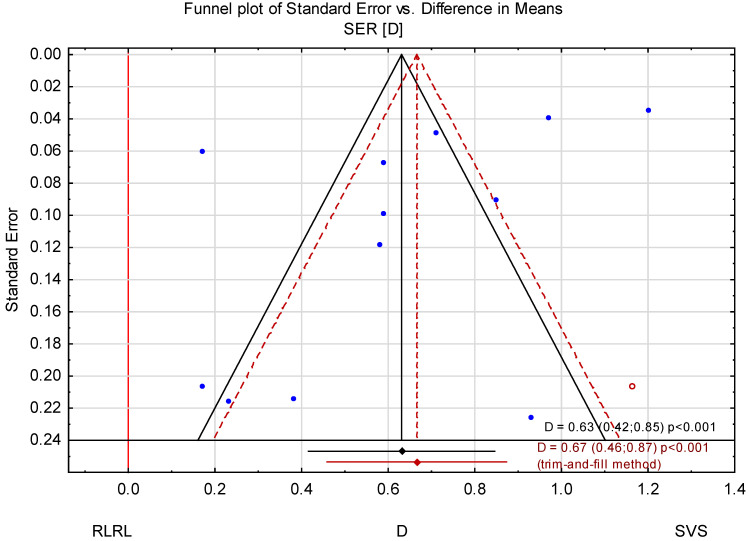
Funnel plot before and after applying the trim-and-fill method (SER).

The trim-and-fill method was used to estimate the effect of potentially missing studies due to publication bias in the funnel plot (Figure 8). After trimming the studies by Zhou L. et al. [32] and Dong et al. [33], the mean change in AL was −0.31 mm (95% CI from −0.36 to −0.27). Using the trim-and-fill method, the imputed point estimate is −0.28 mm (95% CI from −0.33 to −0.22). After using the trim-and-fill method, the mean change in SER was 0.67 D (95% CI from 0.46 to 0.87) (Figure 9 and Figure 10).

### 3.4. Time Points: t = 6 Months and t = 12 Months

**Figure 11:**
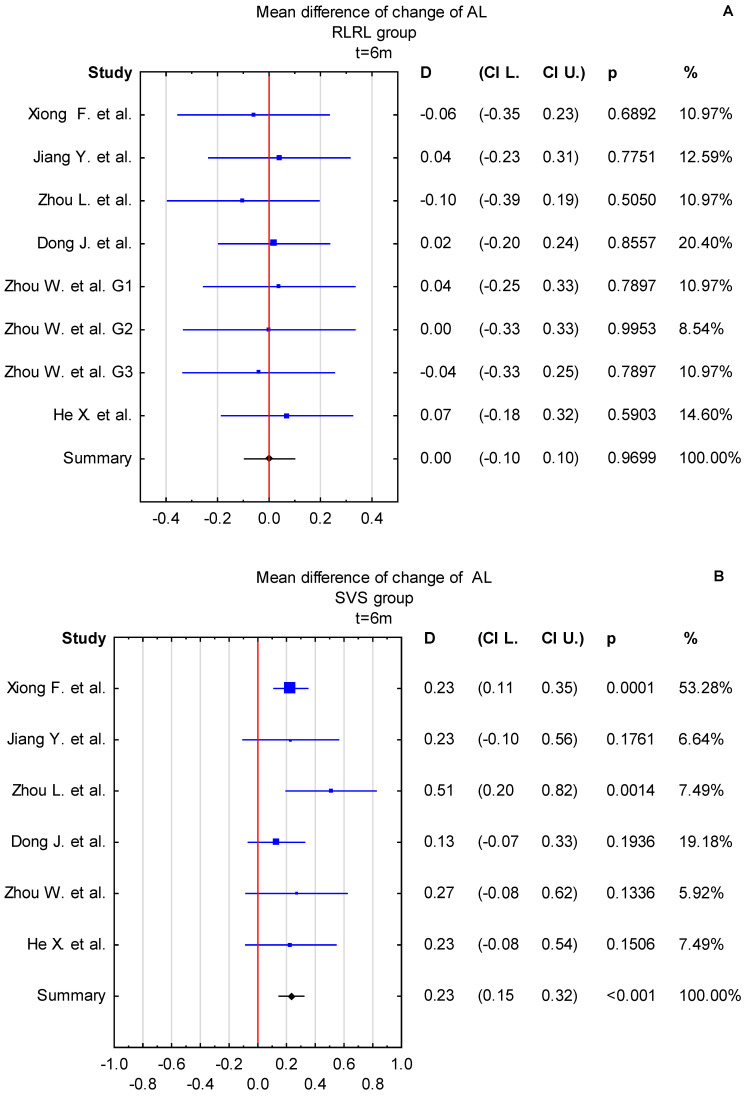

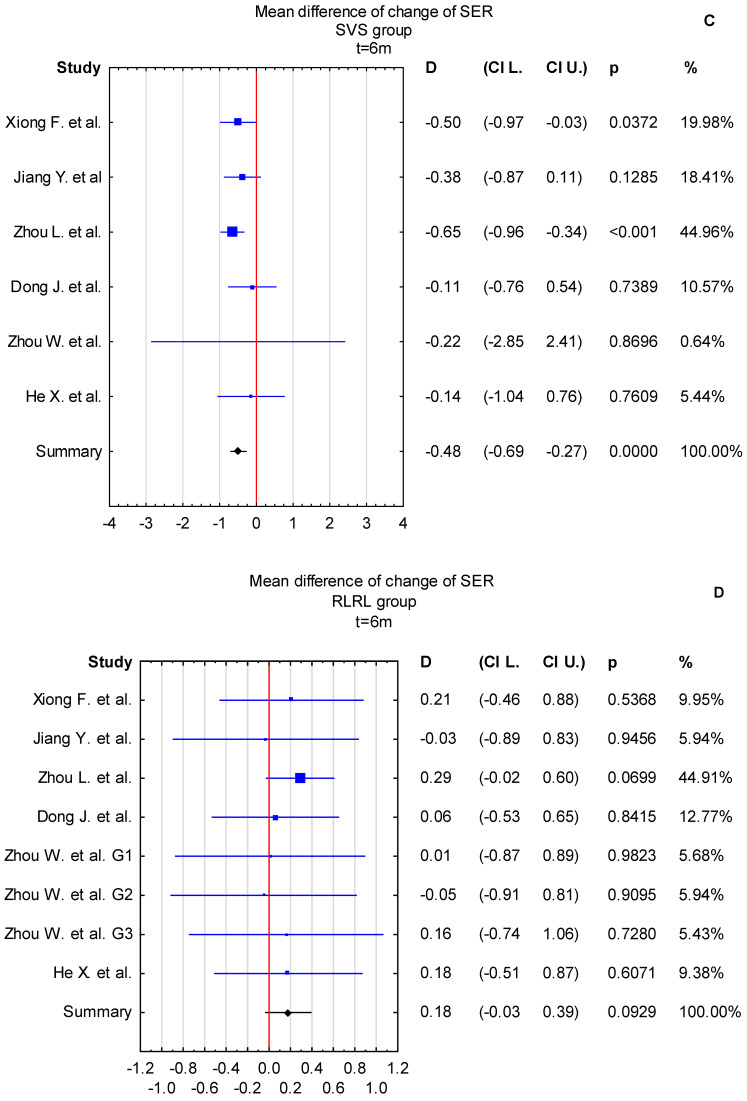

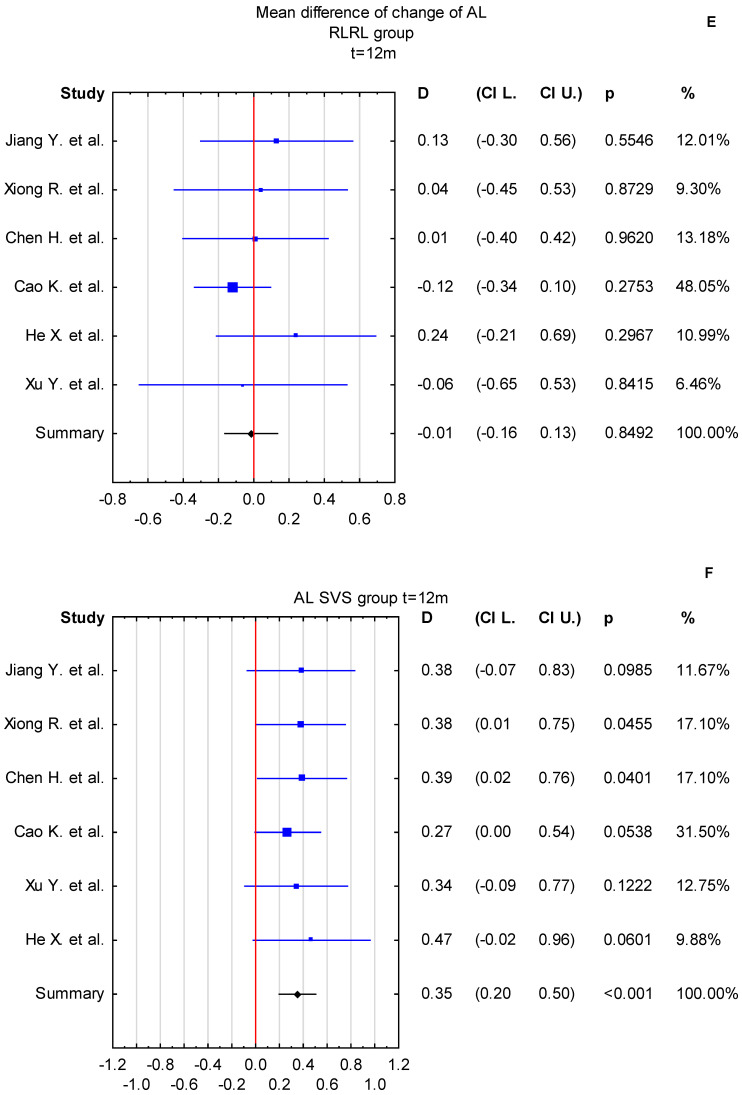

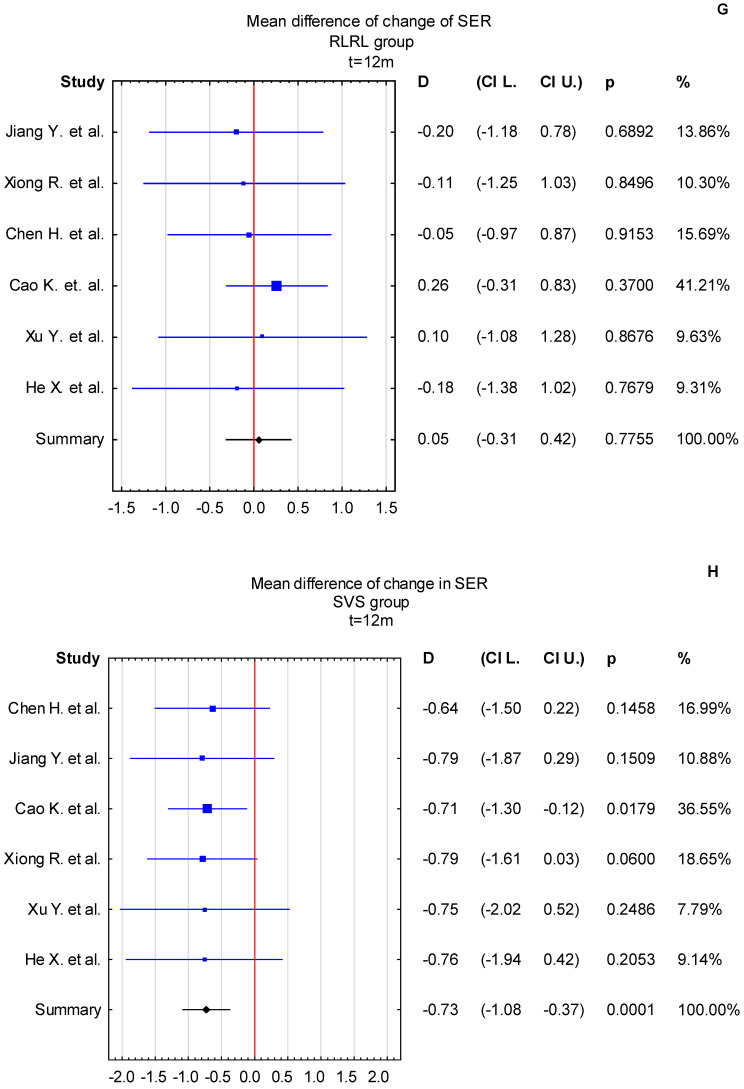
(**A**–**H**) Forest plot of mean difference of change in AL and SER between the RLRL group and SVS group, with *p*-value indicating level of statistical significance. The size of the box represents the point estimate for each study in the forest plot and is proportional to that study’s weight-estimate contribution to the summary estimate. Horizontal lines represent 95% CI. (A–D for t = 6 m and E–H for t = 12 m [30,31,32,33,34,35,36,37,38,39].

Based on the conducted subgroup analysis, the time period of observations plays an important role in assessing changes in myopia. Consequently, an independent meta-analysis was performed separately for the t = 6 months and t = 12 months time points for both the RLRL and SVS groups. The mean difference in AL change at t = 6 months for the RLRL and SVS groups was 0.00 mm (95% CI: −0.10 to 0.10 mm) and 0.23 mm (95% CI: 0.15 to 0.32 mm), respectively. At t = 12 months, the mean difference in AL change for the RLRL and SVS groups was −0.01 mm (95% CI: −0.16 to 0.13 mm) and 0.35 mm (95% CI: 0.20 to 0.50 mm), respectively (Figure 11A,B,E,F). The mean difference in SER change at t = 6 months for the RLRL and SVS groups was 0.18 D (95%CI: −0.03 to 0.39 D) and −0.48 D (95% CI: −0.69 to −0.27 D, respectively. At t = 12 months, the mean difference in SER change for RLRL and SVS groups was 0.05 (95% CI −0.31 to 0.42 D), and −0.73 D (95% CI: −1.08 to −0.37D), respectively (Figure 11C,D,G,H). Statistically significant changes were observed only in the SVS group, at both t = 6 months and t = 12 months. The effect of apparent myopia regression (AL shortening and positive difference in SER change) is caused by the thickening of the retinal choroid layer, which is considered one of the substantial factors affecting myopia progression and growth of the eyeball [55].

## 4. Discussion

This review aimed to demonstrate that RLRL therapy may be effective in slowing myopia progression and delaying axial elongation in a large population of myopic children. RLRL therapy is becoming increasingly prevalent in clinical practice, largely due to its child-friendly nature and the feasibility of home use. As no formal studies on RLRL therapy for myopia management in children were reported before 2019, the available follow-up data are typically limited to six months. In this meta-analysis, we included studies with follow-up periods of up to 24 months, allowing for an expanded understanding of RLRL therapy’s long-term effects. Additionally, as recommended in the “Expert Consensus on Repeated Low-Level Red Light as an Alternative Treatment for Childhood Myopia” in 2022 [56], we included studies that explored varying device power levels. However, due to a limited number of studies investigating different device powers, we were unable to assess the impact of varying intensities on treatment efficacy.

Regarding selection for the meta-analysis, the study of Chen Y. et al. [41] was not considered, as the methodology was different. Here, the child was randomly assigned to either the RLRL group or 0.01%-atropine-eye-drops group.

The reported changes in AL, as well as SER in children with myopia, varied between the different studies. This could result from differences in the age groups of children included in analyses. For example, Yan Xu et al. [37] examined children aged from 6 to 16 years, while He X. et al. [39] included children aged from 6 to 11 years, and Jiang et al. [31] focused on those aged from 8 to 13 years. Additionally, the inclusion criteria regarding SER differed between studies. For instance, Xiong F. et al. [30] and Dong J. et al. [33] included participants with SER less than −0.5 diopters, while He et al. [39] analyzed the more myopic eye in the range from −0.5 D to 0.5 D (in pre-myopia) if at least one parent had SER ≤ −3.00 D, and Cao K. et al. [36] considered both eyes with SER ranging from −6 D to 3 D. Moreover, the duration of red-light therapy varied across studies, ranging from 6 months to 24 months [34]. While the treatment schedule in the included studies was consistent, at 3 min per session and two sessions per day with a minimum interval of 4 h, the number of treatment days per week differed between studies. Xiong R. et al. [34] reported five treatment days per week, whereas Chen H. et al. [35] reported seven. Additionally, although most studies in the provided meta-analysis focus solely on the right eye, Dong et al. [33] included both eyes, considering the mean ocular parameters. Furthermore, the study by Zhou L. et al. [32] included children aged from 4 to 14 years, categorizing them into two groups: preschool children (4–7 years) and school-age children (8–14 years). For our meta-analysis we included only data from the school-aged-children group.

The study by Zhou et al. [38] provides important insights into the efficacy of RLRL therapy, particularly in its effects on SER and axial length (AL). When examining the mean difference in the change in SER between the RLRL therapy group and the SVS group, the inclusion of zero in the 95% confidence interval (CI) at different power levels (0.37 mW, 0.60 mW, and 1.20 mW) suggests a lack of significant effect for RLRL therapy on SER. This indicates that, at least for SER, RLRL therapy might not have a clear and consistent impact. However, the study also highlights a trend where the highest mean difference in SER occurred at the 1.20 mW power level, with a 95% CI ranging from −0.04 to 0.80 (D). The inclusion of zero in the CI suggests that further investigation is required to confirm whether this power output is effective. In contrast, the analysis for AL showed a consistent effect of RLRL therapy, as the CI for axial length did not include zero in any of the included studies. This suggests that RLRL therapy had a reliable impact on axial length, regardless of the power level, which may be an important finding in terms of understanding the overall effectiveness of RLRL therapy in altering eye growth or refractive development.

Additionally, there were differences in red-light-source parameters. The central wavelength was used in the range 630–650 nm, but detailed technical data concerning spectral width and illumination characteristics were not clear. The authors usually omitted detailed analysis of the retinal illumination, which could affect the results and could potentially exceed the safety limits, especially since the number of devices is still growing [47].

In most cases, the power in the pupil plane, wavelength with bandwidth, and manufacturer/model were supported. However, it should be noted that Xiong R. et al. [34], Cao K. et al. [36], and He X. et al. [39] did not explicitly report the power of the devices, while Dong J. et al. [33] omitted the wavelength.

There are limitations to this study. In particular, all of the studies were conducted in China. There is good evidence that ocular parameters, including those concerning the retina, such as retinal capillary density, foveal morphology, or foveal avascular zone, are dependent on ethnicity [57,58]. Additionally, no clear explanation of the mechanism underlying myopia progression control was proposed. There is still a lack of research that could support a hypothesis about the efficacy of the LRLR therapy outside China.

The long-term safety and effectiveness of RLRL therapy require further investigations. Future studies should aim to determine the optimal dose, treatment duration, and device power, as well as regression and possible rebound following RLRL therapy.

## 5. Conclusions

In summary, this study indicates a significant difference in AL and SER between children who wore single-vision distance spectacles (SVS) throughout the study (control group) and those who wore SVS lenses and underwent RLRL treatment (study group). Based on the provided analysis, we confirmed the effectiveness of RLRL therapy in slowing myopia progression and delaying axial elongation in children.

## Figures and Tables

**Table 1:** Characteristics of the included studies, SVS—control group, RLRL—study group.

	Time	SVS	SVS	SVS	SVS Age	RLRL	RLRL	RLRL	RLRL Age	Study Design
	[Months]	AL Change ± SD or (95% CI) [mm]	SER ± SD or (95% CI) [D]	Number of Subjects	Mean ± SD Range [Years]	AL Change ± SD or (95% CI) [mm]	SER ± SD or (95% CI) [D]	Number of Subjects	Mean ± SD Range [Years]	
Xiong F. et al. [30]	1	0.02 ± 0.02	−0.07 ± 0.11	74	10.33 ± 2.03 (7−14)	−0.05 ± 0.07	0.11 ± 0.17	74	10.22 ± 2.38 (7–15)	randomized controlled trial
	3	0.10 ± 0.04	−0.24 ± 0.16			−0.07 ± 0.12	0.22 ± 0.32			
	6	0.23 ± 0.06	−0.50 ± 0.24			−0.06 ± 0.15	0.21 ± 0.34			
Jiang Y. et al. [31] CI	1	0.02 (0.01 to 0.03)	−0.01 (−0.06 to 0.03)	129	10.5 (8−13)	−0.004 (−0.05 to −0.03)	0.08 (0.04 to 0.13)	117	10.4 (8–13)	randomized, parallel-group, single-blind clinical trial
	3	0.10 (0.09 to 0.12)	−0.18 (−0.27 to −0.13)			−0.01 (−0.03 to 0.00)	0.07 (0.02 to 0.12)			
	6	0.23 (0.20 to 0.26)	−0.38 (−0.47 to −0.30)			0.04 (0.02 to 0.07)	−0.03 (−0.11 to 0.05)			
	12	0.38 (0.34 to 0.42)	−0.79 (−0.88 to −0.69)			0.13 (0.09 to 0.17)	−0.20 (−0.29 to −0.11)			
Zhou L. et al. [32]	3	0.33 ± 0.12	−0.47 ± 0.09		8−14	−0.08 ± 0.11	0.07 ± 0.14		8–14	a retrospective case series
	6	0.51 ± 0.16	−0.65 ± 0.16	76		−0.10 ± 0.15	0.19 ± 0.25	76		
	9	0.61 ± 0.20	−0.94 ± 0.13			−0.06 ± 0.18	0.22 ± 0.17			
Dong J. et al. [33]	6	0.13 ± 0.10	−0.11 ± 0.33	55	9.86 ± 1.41 (7–12)	0.02 ± 0.11	0.06 ± 0.30	56	10.3 ± 2.07 (7–12)	randomized, double-blind, controlled clinical trial
Xiong R. et al. [34]	12	0.38 ± 0.19	−0.79 ± 0.42	41	10.79 ± 1.55	0.04 ± 0.25	−0.11 ± 0.58	11	11.18 ± 1.67	multicenter randomized controlled trial
	24	0.64 ± 0.29	−1.24 ± 0.63			0.16 ± 037	−0.31 ± 0.79			
Chen H. et al. [35] CI	12	0.39 (0.33 to 0.45)	−0.64 (−0.78 to −0.51)	40	8.98 ± 1.92 (6−13)	0.01 (−0.05 to 0.07)	−0.05 (−0.08 to 0.19)	46	9.0 ± 1.9 (6−13)	prospective, randomized, controlled study
	12	−0.19 (−0.24 to −0.14)	−0.03 (−0.01 to 0.08)			−0.22 (−0.45 to −0.05)	−0.60 (−0.71 to−0.48)			
Cao K. et al. [36]	12	0.27 ± 0.14	−0.71 ± 0.30	112	9.0 ± 1.9	−0.12 ± 0.11	0.26 ± 0.29	112	9.0 ± 2.0	single-masked, single-center, randomized clinical trial
Yan X. et al. [37] CI	12	0.34 (0.30 to 0.39)	−0.75 (−0.88 to −0.62)	95	11.2 ± 2.1	−0.06 (−0.1 to 0.02)	0.1 (−0.02 to 0.22)	96	10.4 ± 2.4	a prospective, single-blind, parallel-group, multicenter, randomized clinical trial
Zhou W. et al. [38] CI	6	0.27 (−0.50 to 0.30)	−0.22 (−0.50 to 0.30)	43	8.83 ± 1.53 (6–15)	0.04 (−0.01 to 0.08)	0.01 (−0.12 to 0.15)	43	8.51 ± 1.51	single-center, randomized, parallel-group clinical trial
	G1 (0.37 mW)									
	G2 (0.60 mW)					0.00 (−0.05 to 0.05)	−0.05 (−0.18 to 0.07)	47	8.77 ± 1.43	
	G3 (1.20 mW)					−0.04 (−0.08 to 0.01)	0.16 (0.03 to 0.30)	44	8.68 ± 1.39	
He X. et al. [39]	6	0.23 ± 0.16		120	8.3 ± 1.1	0.07 ± 0.13	0.18 ± 0.35	139	8.3 ± 1.1	single-blinded, randomized clinical trial
	12	0.47 ± 0.25				0.24 ± 0.23	−0.18 ± 0.61	32		

CI—publications with confidence intervals given.

**Table 2:** Details of the devices used in publications listed in Table 1.

Manuscript	Power	Wavelength	Manufacturer/Model
Xiong F. et al. [30]	2 ± 0.5 mW	650 nm	Ya Kun Optoelectronic Co. (Wuhan, China)
Jiang Y. et al. [31]	0.29 mW for 4 mm pupil	650 ± 10 nm	Eyerising International—Suzhou Xuanjia Optoelectronics Technology (South Yarra, Australia)
Zhou L. et al. [32]	0.4 mW	635 ± 1 nm	Optoelectronic Co. (Changchun, China)
Dong J. et al. [33]	0.29 mW for 4 mm pupil	n/a	Eyerising International—Suzhou Xuanjia Optoelectronics Technology (South Yarra, Australia)
Xiong R. et al. [34]	Class 1 device for 4 mm pupil	650 ± 10 nm	Eyerising International —Suzhou Xuanjia Optoelectronics Technology (South Yarra, Australia)
Chen H. et al. [35]	0.35 ± 0.02 mW	635 nm	Jilin Londa Optoelectronics Technology (Jilin City, China)/LD-A
Cao K. et al. [36]	n/a	650 nm	Hunan EnVan Technology Co., Ltd. (China)/YF020A
Yan X. et al. [37]	0.29 mW for 4 mm pupil	650 ± 10 nm	Eyerising International —Suzhou Xuanjia Optoelectronics Technology (South Yarra, Australia)
Wen Z. et al. [38]	0.37 ± 0.2 mW (group 1) 0.60 ± 0.2 mW (group 2) 1.2 ± 0.2 mW (group 3)	650 nm	Beijing Ming Ren Shi Kang Science & Technology Co. (Beijing, China)/sky-n1201
He X. et al. [39]	n/a	650 ± 10 nm	Eyerising International —Suzhou Xuanjia Optoelectronics Technology (South Yarra, Australia)

## Data Availability

All data generated or analyzed during this study are included in this published article.
